# Clinical analgesic efficacy of dexamethasone as a local anesthetic adjuvant for transversus abdominis plane (TAP) block: A meta-analysis

**DOI:** 10.1371/journal.pone.0198923

**Published:** 2018-06-14

**Authors:** Qi Chen, Ran An, Ju Zhou, Bin Yang

**Affiliations:** Department of Anesthesiology, Chongqing University Cancer Hospital/Chongqing Cancer Institute, Chongqing, China; Sunnybrook Health Sciences Centre, University of Toronto, CANADA

## Abstract

**Background:**

Perineural dexamethasone has been shown to prolong the duration of local anesthetic (LA) effect in regional anesthesia; however, the use of perineural dexamethasone as an adjuvant to to the transversus abdominis plane (TAP) block remains controversial. This meta-analysis sought to assess the efficacy of dexamethasone in prolonging the TAP block and enhancing recovery after abdominal surgery.

**Methods:**

We identified and analyzed 9 RCTs published on or before September 30, 2017, regardless of the original language, after searching the following 6 bibliographic databases: PubMed, EMBASE, Medline, Springer, Ovid, and the Cochrane Library. databases. These studies compared the effects of perineural dexamethasone mixed with local anesthetic versus local anesthetic alone in the TAP block. The Cochrane Collaboration’s Risk of Bias Tool was used to evaluate the methodological quality of each RCT. The primary outcomes were the time until the first request for postoperative analgesics and the analog pain scores at 2, 6, 12, and 24 h after surgery. The secondary outcomes were the analgesic consumption and the incidence of nausea and vomiting on the first day after surgery. We used Trial Sequential Analysis (TSA) to control for random errors.

**Results:**

Perineural dexamethasone prolonged the duration of LA effect in the TAP block [mean difference (MD): 2.98 h; 95% confidence interval (CI): 2.19 to 3.78] and reduced analog pain scores at 2 h [MD: -1.15; 95% CI: -2.14 to -0.16], 6 h [MD: -0.97; 95% CI: -1.51 to -0.44], and 12 h [MD: -0.93; 95% CI: -1.14 to -0.72] postoperatively. Furthermore, the use of perineural dexamethasone was associated with less analgesic consumption [standard mean difference: -1.29; 95% CI: -1.88 to -0.70] and a lower incidence of nausea and vomiting [odds ratio: 0.28; 95% CI: 0.16 to 0.49] on the first day after surgery.

**Conclusion:**

Dexamethasone prolongs the LA effect when used as an adjuvant in the TAP block and improves the analgesic effects of the block.

## Introduction

Safe and effective postoperative analgesia is important for enhancing recovery after surgery; however, severe pain after abdominal surgery remains a significant problem. Furthermore, spinal anesthesia or use of systemic opioid analgesia can result in adverse effects such as nausea, vomiting, pruritus, and respiratory depression [1–3]. As part of a multimodal analgesic regimen, a peripheral nerve block can decrease opioid consumption, providing more effective analgesia with fewer adverse effects [2–4]. With the increasing use of ultrasound guidance during peripheral nerve blocks, truncal blocks, such as the transversus abdominis plane (TAP) block, are now widely used for analgesia after abdominal surgery [5–7].

The TAP block, which was first described by Rafi [8] in 2001, involves injecting local anesthetic (LA) between the T6–T9 spinal nerve roots to block nerve signal conduction and alleviate pain after abdominal surgery. However, the TAP block may not provide a sufficient duration of analgesia. Dexamethasone, a high-potency, long-acting glucocorticoid, has been shown to prolong peripheral nerve blockade in animals [9]. Dexamethasone binds to glucocorticoid receptors and inhibits potassium conductance, which decreases nociceptive C-fiber activity [10, 11]. Dexamethasone may also extend the duration of analgesia via local vasoconstrictive and systemic anti-inflammatory effects [11, 12].

A number of systematic reviews and meta-analyses have confirmed the efficacy of dexamethasone for prolonging the duration of peripheral nerve blocks [13–15]. More specifically, dexamethasone provides better analgesic efficacy and decreased analgesic consumption postoperatively compared with LA alone. However, recent well-designed randomized controlled trials (RCTs) have failed to show statistically significant prolongation of LA effects when dexamethasone was used as an adjuvant to the TAP block [16, 17]. Thus, we sought to determine the analgesic efficacy of dexamethasone as an adjuvant to the TAP block. The primary outcomes of this meta-analysis were the time until the first request for postoperative analgesics and analog pain scores at 2, 6, 12, and 24 h after surgery.

## Methods

We conducted a systemic review and meta-analysis in accordance with the Preferred Reporting Items for Systematic Reviews and Meta-analysis (PRISMA) guidelines [18](S1 File). The protocol of this systemic review was not registered.

### Literature search

We searched for studies published on or before September 30, 2017 that evaluated the efficacy of dexamethasone as an adjuvant to TAP blocks using the following 6 bibliographic databases: PubMed, EMBASE, Medline, Ovid, Springer, and the Cochrane Library. The comprehensive search terms (key words, phrases, and medical subject headings) were as follows: transversus abdominis plane block, peripheral nerve block, or regional anesthesia; dexamethasone, glucocorticoids, or steroid; and analgesia postoperative, postpartum period, or post-extraction. The following keyword fragments were also included: Echo*, Ultrasound*, Analg*, Anesth*, Adjuvants*.

### Eligibility criteria

We searched for complete articles and published abstracts of RCTs comparing the effects of the TAP block with or without perineural dexamethasone. We included studies that used the TAP block for postoperative analgesia, regardless of the type of abdominal surgical procedures or patient age. The search was not limited by language of the articles or by the type or dose of LA.We excluded RCTs comparing dexamethasone with other adjuvants.

### Trial selection

**Fig 1:**
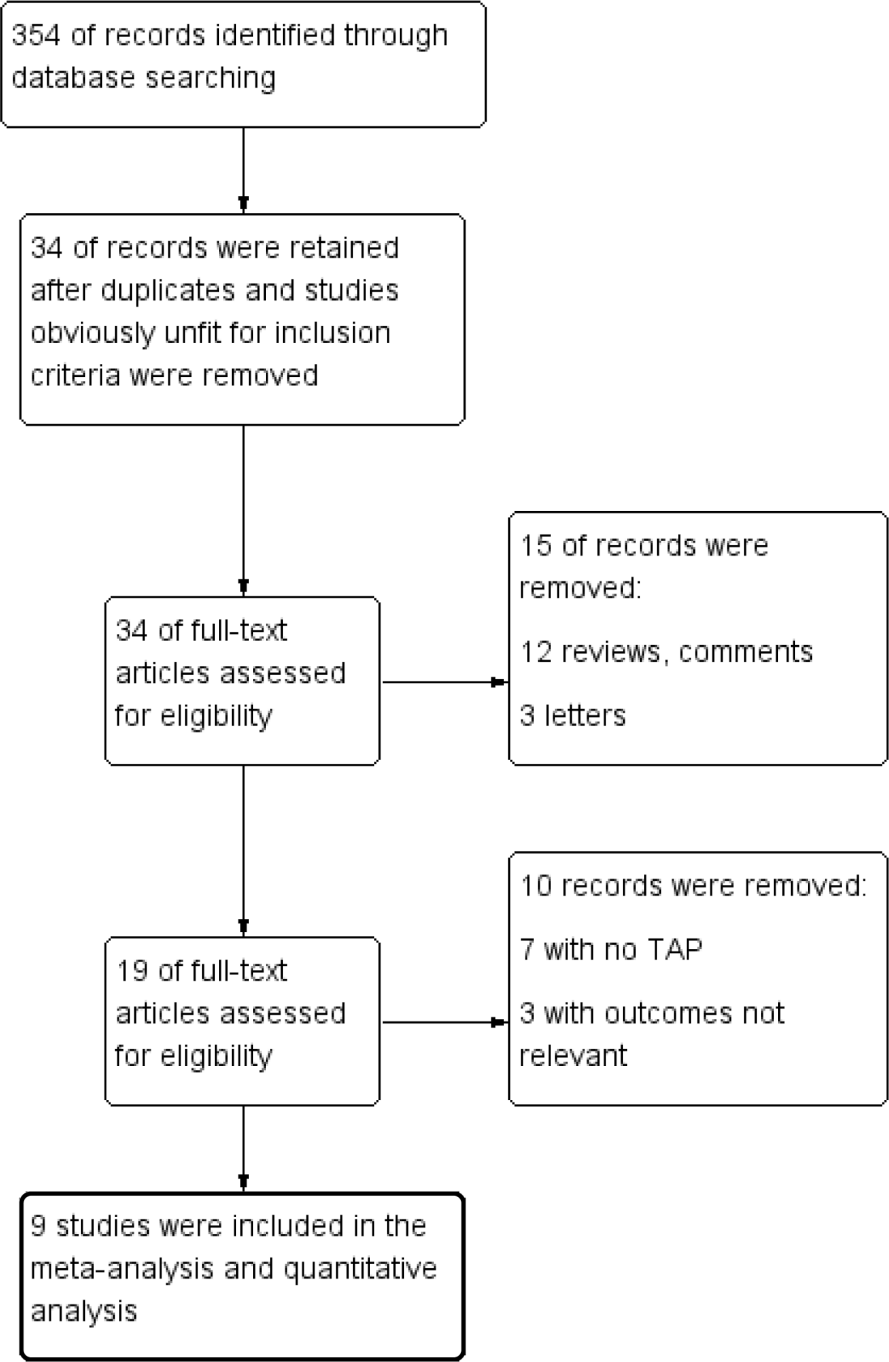
PRISMA flow diagram. PRSIMA, Preferred Reporting Items for Systematic Reviews and Meta-Analyses.

Two authors (Q.C. and R.A.) separately evaluated the abstracts retrieved. The decision to include qualifying studies was made by consensus between these 2 authors. The opinion of a third author (B.Y.) was obtained when agreement could not be reached. The results of the trial selection process are presented in the PRISMA flowchart (Fig 1).

### Risk of bias assessment

**Fig 2:**
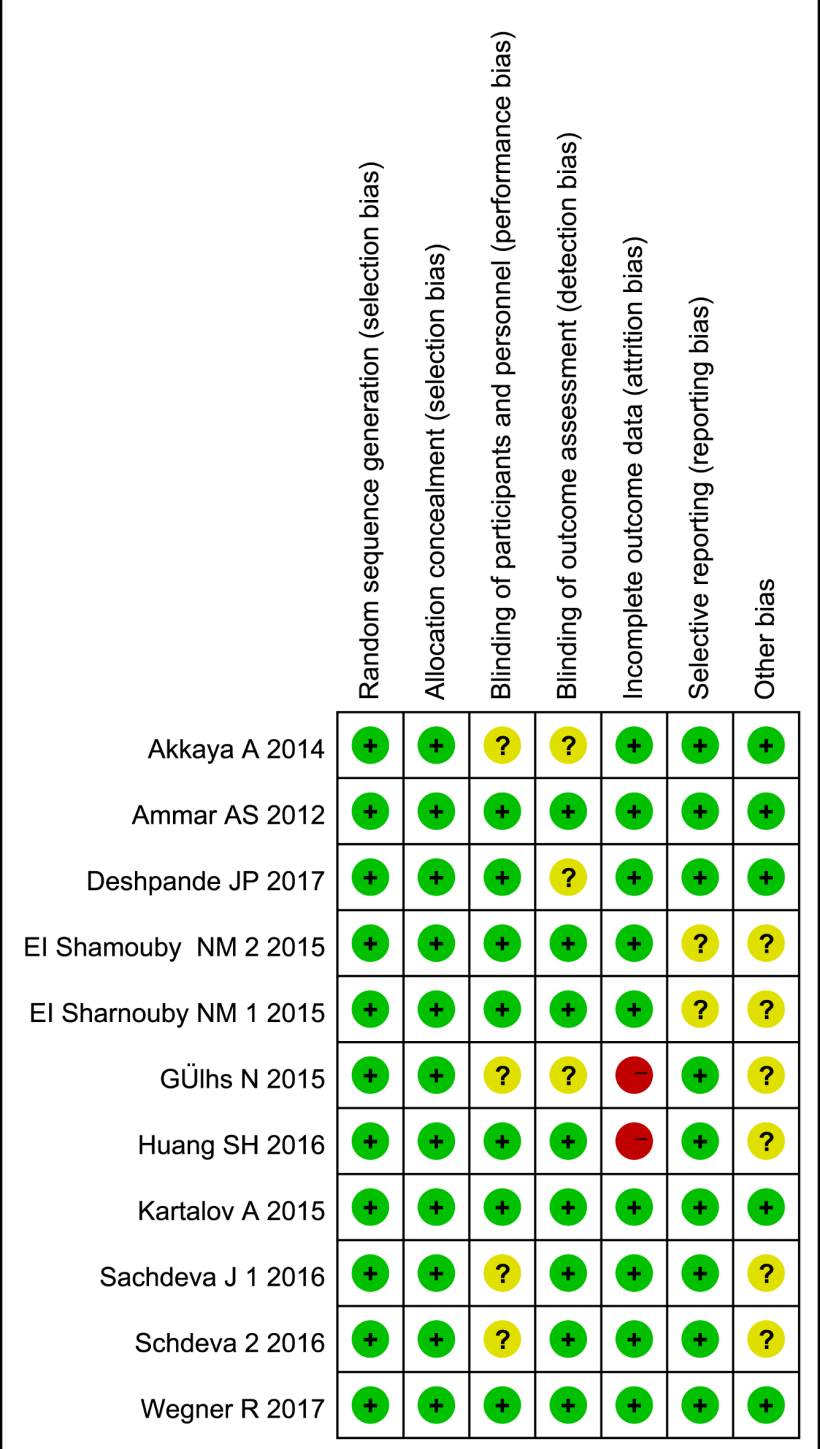
Evaluation of risk of bias for each included study. Green circle indicates low risk of bias, red circle indicates high risk of bias, yellow circle indicates unclear risk of bias.

The quality of each RCT was independently assessed by 2 authors (Q.C. and R.A.) using the Cochrane Collaboration Risk of Bias Tool [19]. This tool evaluates RCTs for various types of bias, including selection, performance, detection, attrition, and reporting bias(Fig 2). A quality score was assigned to each RCT by consensus between the 2 authors, and a third author (B.Y.) was consulted when an agreement could not be reached. RCT quality scores were not a factor for trial exclusion. The risk of publication bias also assessed using the Egger test.

### Data extraction

Data were independently extracted by 2 authors (Q.C. and R.A.). Discrepancies in data extraction were resolved by review and discussion. The opinion of a third author (B.Y.) was sought if consensus could not be reached. These extracted data included the following: name of the principal author, publication year, type of surgery and anesthesia, number of patients in each group, and details regarding the TAP block (LA type and dose, dexamethasone dose). We also extracted data regarding duration of the block, pain scores, analgesic consumption, nausea and vomiting, and adverse effects (Table 1).

**Table 1:** Trial characteristics.

Study	year	No. patients	Surgery/Anesthesia	Gender	TAP block local Anesthetic	Time^2^	Multimodal analgesia	outcomes
Akkaya A [27]	2014	21/21^1^	Cesarean section/Spinal	female	0.25% levobupivacaine 30ml+NS 2ml/ 0.25%levobupivacaine30ml+8mgDXM 2ml	after	tramadol	analgesic consumption, time of first request and PONV
Ammar AS [28]	2012	30/30	open abdominal hysterectomy/General	female	0.25%bupivacaine20ml+0.9%saline2ml 0.25% bupivacaine 20ml+8mg DXM 2ml	before	morphine and acetaminophen	analgesic consumption, time of first request, PONV and pain score
Deshpande JP [30]	2017	30/30	open abdominal hysterectomy/spinal	female	0.5%ropivacaine20ml+0.9%saline1ml/ 0.5% ropivacaine 20ml+4mgDXM 1ml	after	Paracetamol and tramadol	analgesic consumption, time of first request, PONV and pain score
EI Shamouby NM [29]	2015	33/34/34	Laparoscopic vertical banded Gastroplasty/general	both	0.25% bupivacaine20ml+0.9%/0.25%bupivacaine20ml+4mgDXM/0.25%bupivacaine20ml+8mg DXM	after	Paracetamol and meperidine	analgesic consumption, time of first request, PONV and pain score
GÜlhs N [26]	2015	30/30	Openabdominal hysterectomy/general	female	0.25% bupivacaine 19ml+0.9% saline 1ml 0.25% bupivacaine 19ml+4mg DXM 1ml	after	morphine	analgesic consumption, time of first request and PONV
Huang SH [16]	2016	20/20	Laparoscopic cholecystectomy/general	both	0.375%ropivacaine15ml/ 0.375% ropivacaine +5mgDXM 15ml	before	parecoxib andsulfentanil	analgesic consumption,time of first request, PONV and pain score
Kartalov A [24]	2015	30/30	inguinal herniarepair/general	both	0.5% ropivacaine25ml/ 0.5% ropivacaine +4mgDXM 25ml	before	Morphine andparacetamol	analgesic consumption and pain score;
Sachdeva J [25]	2016	35/35	Cesarean section/spinal	female	0.2% ropivacaine20ml/ 0.2% ropivacaine +4mgDXM 20ml	after	Diclofenac andtramadol	analgesic consumption, time of first request and PONV
Wegner R [17]	2017	41/41	Heriarepair Spermatocelectomy/general	both	0.2% ropivacaine20ml/ 0.2% ropivacaine+8mgDXM 20ml	after	Oralpain medication	PONV and pain score

1: Control group/Dexamethasone group

2:Timing of TAP block relative to Surgery

DXM: Dexamethasone

PONV: Postoperative nausea and vomiting

Among the studies that met our selection criteria, some did not show the results as a mean and standard deviation (SD) or standard error of the mean and 95% confidence interval (CI). In such cases, we e-mailed the study authors twice requesting the original data. If there was no reply, the mean was considered to be equivalent to the median and the SD was approximated to be the interquartile range divided by 1.35 or the range divided by 4 [19, 20]. For quantitative analysis, pain scores that were recorded using the visual analog scale (VAS) were transformed to a standardized 0–10 analog scale (0 = no pain and 10 = worst pain imaginable) [21]. Because different drugs were used postoperatively, the standard mean difference (SMD) was used to compare postoperative analgesic consumption.

### Outcomes

The primary outcomes of this meta-analysis were the time until the first request for postoperative analgesics and the NRS pain score at 2, 6, 12, and 24 h after surgery. The secondary outcomes were the analgesic consumption and the incidence of nausea and vomiting on the first day after surgery.

### Statistical analysis

Revman 5.3 software was used for all statistical analyses. The effect size for continuous data are expressed as the mean difference (MD) or SMD and the 95% CI. The effect size for dichotomous data are expressed as the odds ratio (OR) and the 95% CI. The χ^2^ test P-value and the I^2^ value were used to determine the level of heterogeneity. A random effect model was used in cases of heterogeneity (P<0.1 or I^2^≥50%), and a fixed effect model was used in cases of homogeneity (P≥0.1 or I^2^<50%) [22]. Publication bias was analyzed using the Egger test, whereby P>0.05 indicated no statistically significant publication bias.

Trial Sequential Analysis(TSA) depends on the quantification of the required information size. We calculated a diversity-adjusted (D2) required information size, since the heterogeneity adjustment with I2 underestimates the required information size and estimated the required information size using 0.05 for type 1 error,0.20 for type 2 error. The relative risk reduction from the control group event rate from low-bias-risk trials included in the meta-analysis, according to the TSA user manual [23]. We also performed sensitivity analyses with a power of 80% and assuming a 20% RRR.

## Results

### Literature search

The literature search identified 354 studies, including 9 qualifing RCTs with a total of 575 patients that met the inclusion criteria (Fig 1) [16, 17, 24–30]. The characteristics of patients and interventions are summarized in Table 1. All 9 RCTs compared the TAP block using LA with dexamethasone versus LA alone. All studies used a long-acting LA for the TAP block, including bupivacaine in 3 studies [26, 28, 29], ropivacaine in 5 studies [16, 17, 24, 25, 30], and levobupivacaine in 1 study [27].

### Study quality

Seven studies did not have a high risk of bias for any of the evaluated criteria. Two studies had a high risk of attrition bias, as well as several elements representing an unclear risk of bias (Fig 2).

### Time until first analgesic request

**Fig 3:**
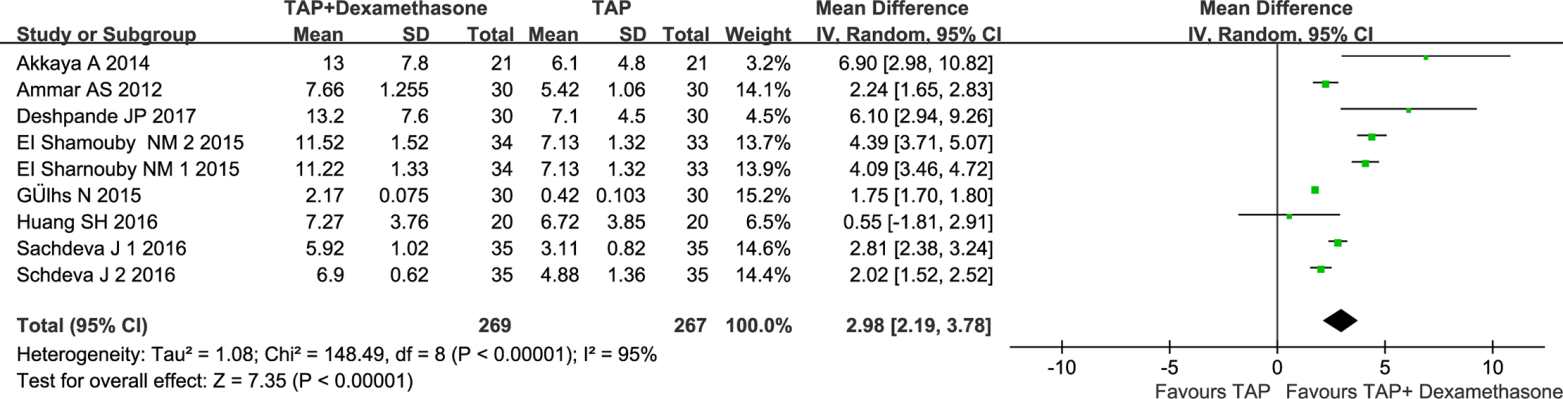
Effect of perineural dexamethasone on duration of analgesia when combined with local anesthetics.

The duration of the TAP block was reported in 7 of the 9 included studies, (n = 433 patients of which 234 received perineural dexamethasone), which was defined as the time to first analgesic request after surgery [16, 25–30]. On average, dexamethasone prolonged the block duration by 2.98 h (95% CI: 2.19 to 3.78; I^2^ = 95%; P<0.00001) from a baseline of 5.34 h without dexamethasone; however, there was a large degree of heterogeneity(Fig 3). The Egger test for publication bias (P = 0.005) and sensitivity analysis did not significantly alter the summarized results. And TSA indicated that the sample size in the meta-analysis was larger than the required sample size (n = 315)(S1 Fig).

### Pain scores and analgesic consumption postoperatively

**Fig 4:**
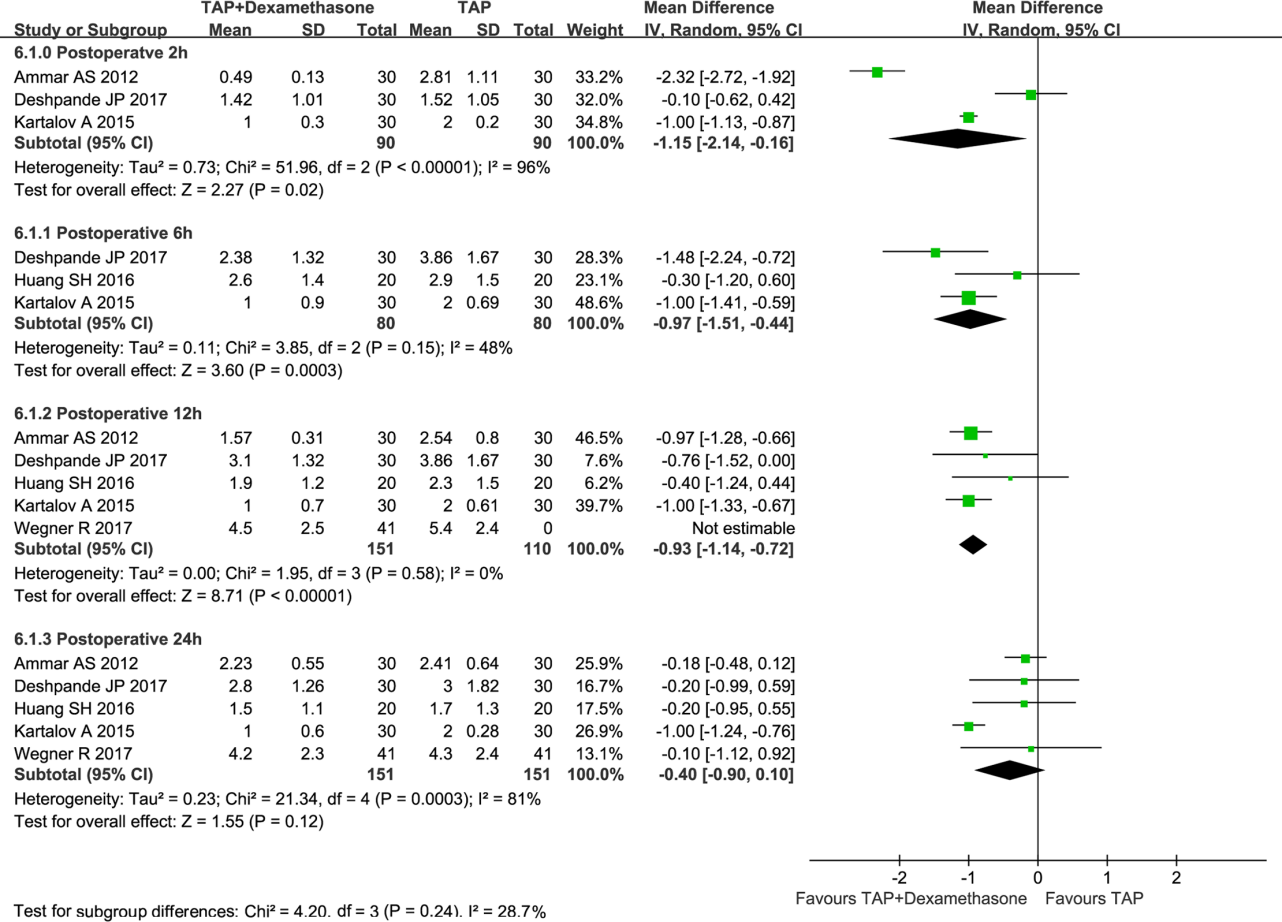
Effect of perineural dexamethasone on analog pain scores at 2, 6, 12, and 24 h after surgery when combined with local anesthetics.

Eight of the 9 studies (n = 505 patients) measured pain scores postoperatively, and scores taken at 2, 6, 12, and 24 h after surgery were used for this meta-analysis [16, 17, 24, 26–30]. The use of dexamethasone reduced analog pain scores by an average of 1.15 points at 2 h (n = 180; 95% CI: -2.14 to -0.16; I2 = 96%; P = 0.02), 0.97 points at 6 h (n = 160; 95% CI: -1.51 to -0.44; I2 = 48%; P = 0.0003), and 0.93 points at 12 h postoperatively (n = 302;95% CI: -1.14 to -0.72; I^2^ = 0%; P<0.00001). There was no statistically significant difference in pain scores at 24 h postoperatively (n = 302; 95% CI: -0.90 to 0.10; I^2^ = 81%; P = 0.12)(Fig 4). The Egger test for publication bias (P = 0.070 at 2 h postoperatively; P = 0.311 at 6 h postoperatively; P = 0.552 at 12 h postoperatively; and P = 0.215 at 24h postoperatively) and sensitivity analysis did not significantly alter the summarized results. TSA results demonstrated that the cumulative Z-score of VAS at 6h, 12 h and 24 h crossed its monitoring boundaries and reliable conclusions had been drawn. But the sample size of VAS at 2 h did not reach the required sample size(S2–S5 Figs).

**Fig 5:**
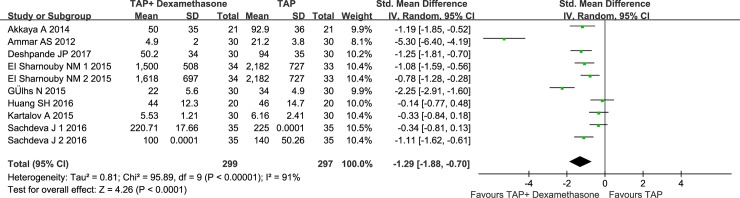
Effect of perineural dexamethasone on analgesic consumption during the first 24 h postoperatively when combined with local anesthetics.

Results describing postoperative analgesic consumption during the first 24 h postoperatively were available from 8 studies (n = 596 patients) [16, 24–30]. Compared with the control group, perineural dexamethasone was effective in reducing postoperative analgesic consumption by -1.29 (95%CI: -1.88 to -0.70; I2 = 91%; P<0.00001)(Fig 5). The Egger test for publication bias (P = 0.215) and sensitivity analysis did not significantly alter the summarized results. And TSA indicated that the sample size in the meta-analysis was larger than the required sample size (n = 557)(S6 Fig).

### Nausea and vomiting

**Fig 6:**
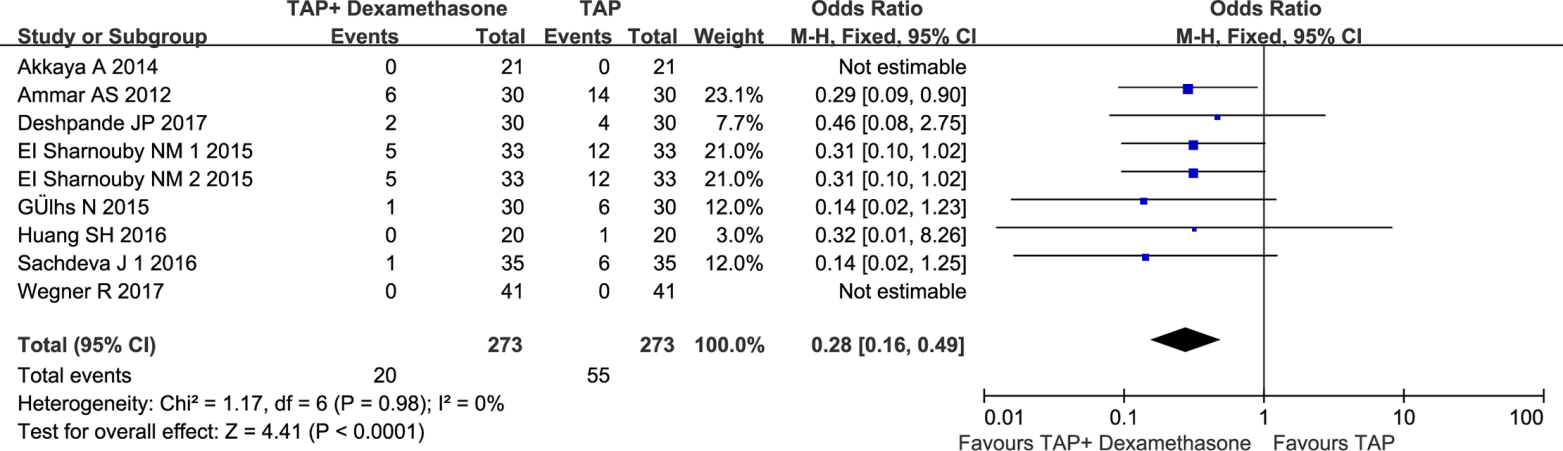
Effect of perineural dexamethasone on nausea and vomiting during the first 24 h postoperatively when combined with local anesthetics.

Eight RCTs (n = 515 patients) investigated the incidence of nausea and vomiting during the first 24 h postoperatively. The incidence of nausea and vomiting was 72% lower in patients who received perineural dexamethasone than in patients who did not receive perineural dexamethasone (95%CI: 0.16 to 0.49; I^2^ = 0%; P<0.00001) [16, 17, 25–30](Fig 6). The Egger test for publication bias (P = 0.072) and sensitivity analysis did not significantly alter the summarized results. And TSA indicated that the sample size in the meta-analysis was larger than the required sample size (n = 175)(S7 Fig).

### Complications

None of the patients in the 9 RCTs had block-related complications, such as bleeding, infection, or abdominal cavity penetration, nor were neurological complications reported in any of the RCTs included in this meta-analysis.

## Discussion

This is the first meta-analysis to evaluate the analgesic efficacy of perineural dexamethasone when used as an adjuvant to the TAP block. After analyzing the combined results of 9 RCTs, we found that the use of perineural dexamethasone significantly prolongs the duration of sensory blockade. Furthermore, we found a modest reduction in pain scores at 2, 6, and 12 h postoperatively, as well as a reduction in the 24-h analgesic consumption when perineural dexamethasone was used as an adjuvant to the TAP block. Therefore, this meta-analysis indicates that dexamethasone, used as an adjuvant to the TAP block, can enhance the analgesic effect and prolong the duration of analgesia. However, the clinical significance of our results may be limited by the high degree of heterogeneity found in the included studies.

Our results are consistent with another recent meta-analysis that assessed the efficacy of dexamethasone as an adjuvant to peripheral nerve blocks, which included only 2 RCTs related to the TAP block [15]. Furthermore, a systematic review and meta-analysis assessing dexamethasone as an adjuvant to brachial plexus blocks has been reported, yet the optimal dose remains unclear [31]. Therefore, we performed a broader search and assessed more RCTs concerning use of dexamethasone in the TAP block, and we assessed its efficacy at doses ranging from 4 to 8 mg. Unfortunately, none of the RCTs in this meta-analysis identified an ideal dosage. Moreover, none of these RCTs included pediatric patients. Therefore, further research is needed to establish the appropriate dose of perineural dexamethasone in both adults and children.

In addition, we found a nearly 72% reduction in the incidence of nausea and vomiting when dexamethasone was used in the TAP block compared with LA alone. As previously reported, the TAP block has been associated with an equivalent or a slightly lower incidence of nausea and vomiting compared with standard postoperative analgesic methods, and these benefits are proposed to result from the corresponding reduction in opioid consumption [32–34]. Our results also confirmed a markedly lower incidence of nausea and vomiting when dexamethasone was added to the LA compared with LA alone in the TAP block. Nevertheless, the mechanism underlying this reduction in nausea and vomiting remains unclear.

Although a variety of drugs are used as adjuvants to LA in peripheral nerve blocks, dexamethasone appears to be the best agent for prolonging analgesia and is favored over tramadol, clonidine, fentanyl, and neostigmine [35–38]. Dexmedetomidine has been reported to have a similar efficacy as dexamethasone in terms of duration of postoperative analgesia and reduction in postoperative nausea and vomiting; however, some reports have shown that dexmedetomidine was associated with an increased incidence of hypotension and bradycardia [39, 40]. Thus, dexamethasone is currently considered to be the most ideal adjuvant to LA in peripheral nerve blocks.

We did not find any major complications associated with the use of perineural dexamethasone in our synthesis; however, complications cannot be ruled out and further observational data are needed. Of note, the use of dexamethasone did not increase the risk of neurotoxic complications related to LA. In fact, some *in vitro* murine studies have found that dexamethasone actually weakens the neurotoxicity of bupivacaine at a cellular level [41]. However, the application of perineural dexamethasone remains an off-label route of administration, and physicians should be cautious in administering solutions containing neurotoxic preservatives [15].

This meta-analysis has several limitations that should be considered. First, even after we accounted for the type of surgery, type of anesthesia, dosage of dexamethasone, TAP block technique, and method of postoperative multimodal analgesia, the heterogeneity of the included studies remained high. Second, pooling data for a meta-analysis and converting median and range values into mean and SD values inevitably results in some degree of uncertainty in estimating the effect size. Third, the duration of the TAP block and the time until the first postoperative analgesic was requested are not identical outcome measures. Finally, the funnel plot displayed an obvious asymmetry, indicating the potential for either publication bias, a language bias, inflated estimates due to a defective methodological design in smaller studies, or a lack of publication of small trials with adverse results.

## Conclusion

In conclusion, our meta-analysis shows that dexamethasone used as an adjuvant to LA prolongs the duration of the TAP block and improves its analgesic effects. Further studies are needed to determine the optimal dose of dexamethasone and its potential adverse effects.

## Supporting information

S1 File PRISMA checklist. (DOC)

S1 Fig TSA for sample size of time until first analgesic request. (PNG)

S2 Fig TSA for sample size of pain score at 2h postoperatively. (PNG)

S3 Fig TSA for sample size of pain score at 6h postoperatively. (PNG)

S4 Fig TSA for sample size of pain score at 12h postoperatively. (PNG)

S5 Fig TSA for sample size of pain score at 24h postoperatively. (PNG)

S6 Fig TSA for sample size of analgesic consumption postoperatively. (PNG)

S7 Fig TSA for sample size of incidence of PONV postoperatively. (PNG)
