# Supplementary figures and images for: Clinical analgesic efficacy of dexamethasone as a local anesthetic adjuvant for transversus abdominis plane (TAP) block: A meta-analysis
Source: PLoS One. 2018 Jun 14;13(6):e0198923. doi: 10.1371/journal.pone.0198923 (PMC6002066; doi:10.1371/journal.pone.0198923)

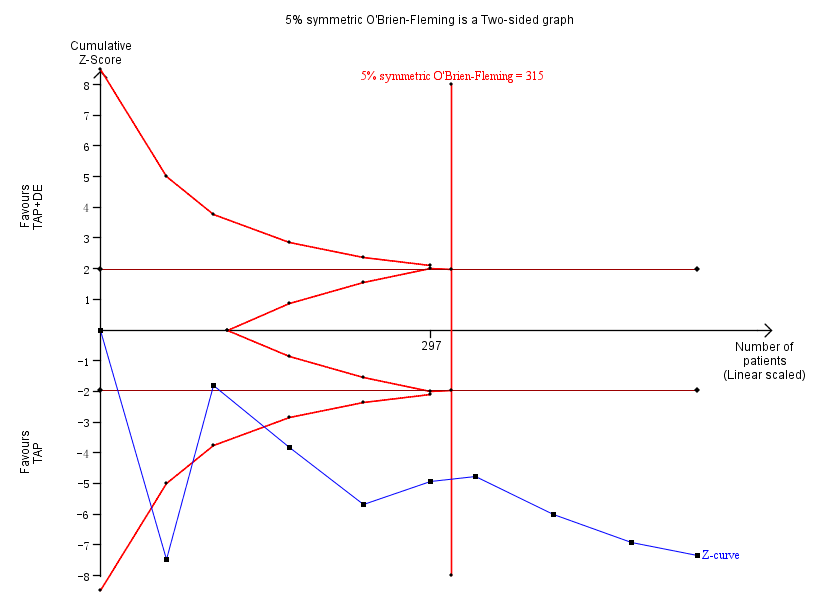

Supplement: S1 Fig — (PNG) [file pone.0198923.s002.png]

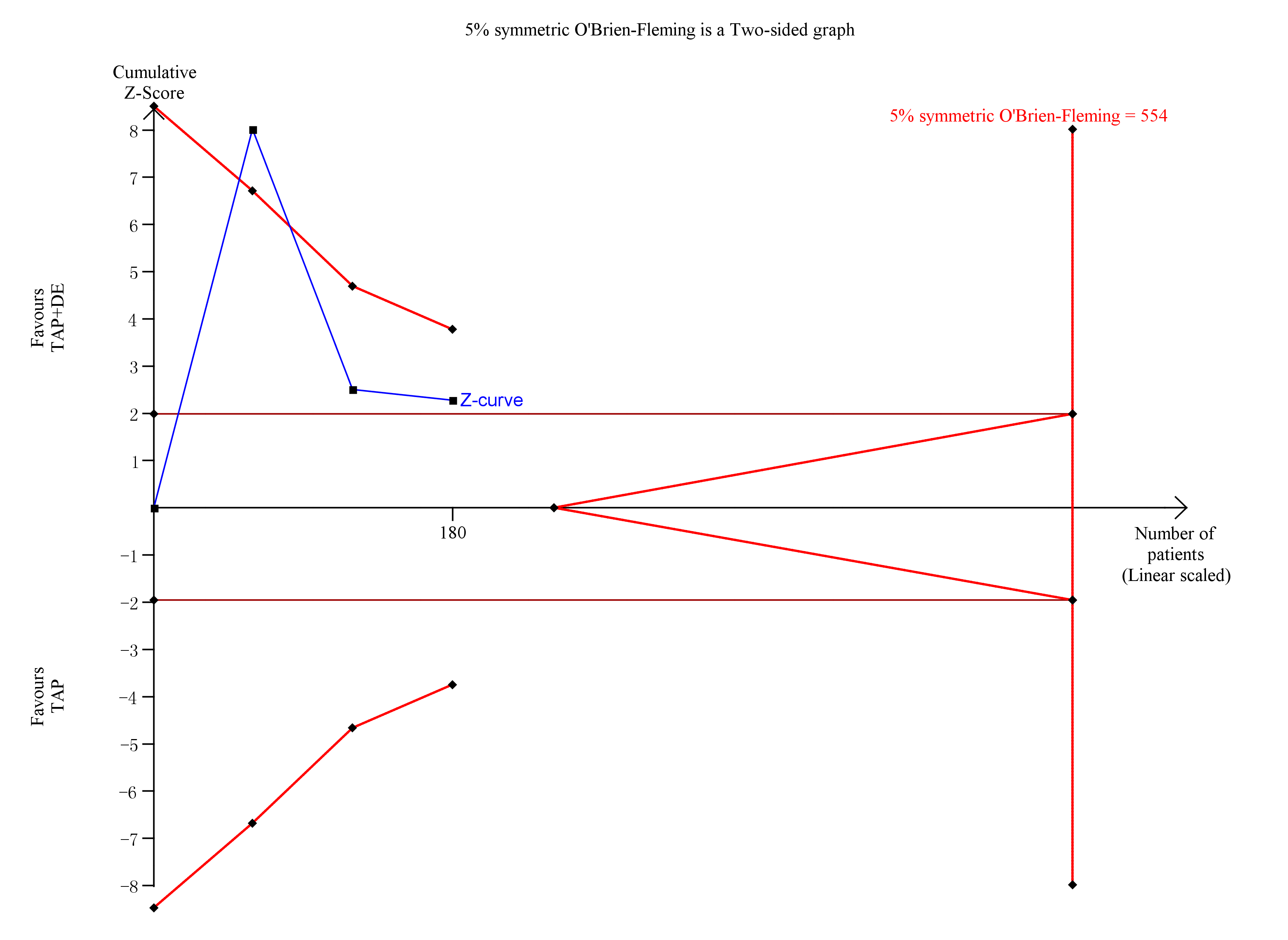

Supplement: S2 Fig — (PNG) [file pone.0198923.s003.png]

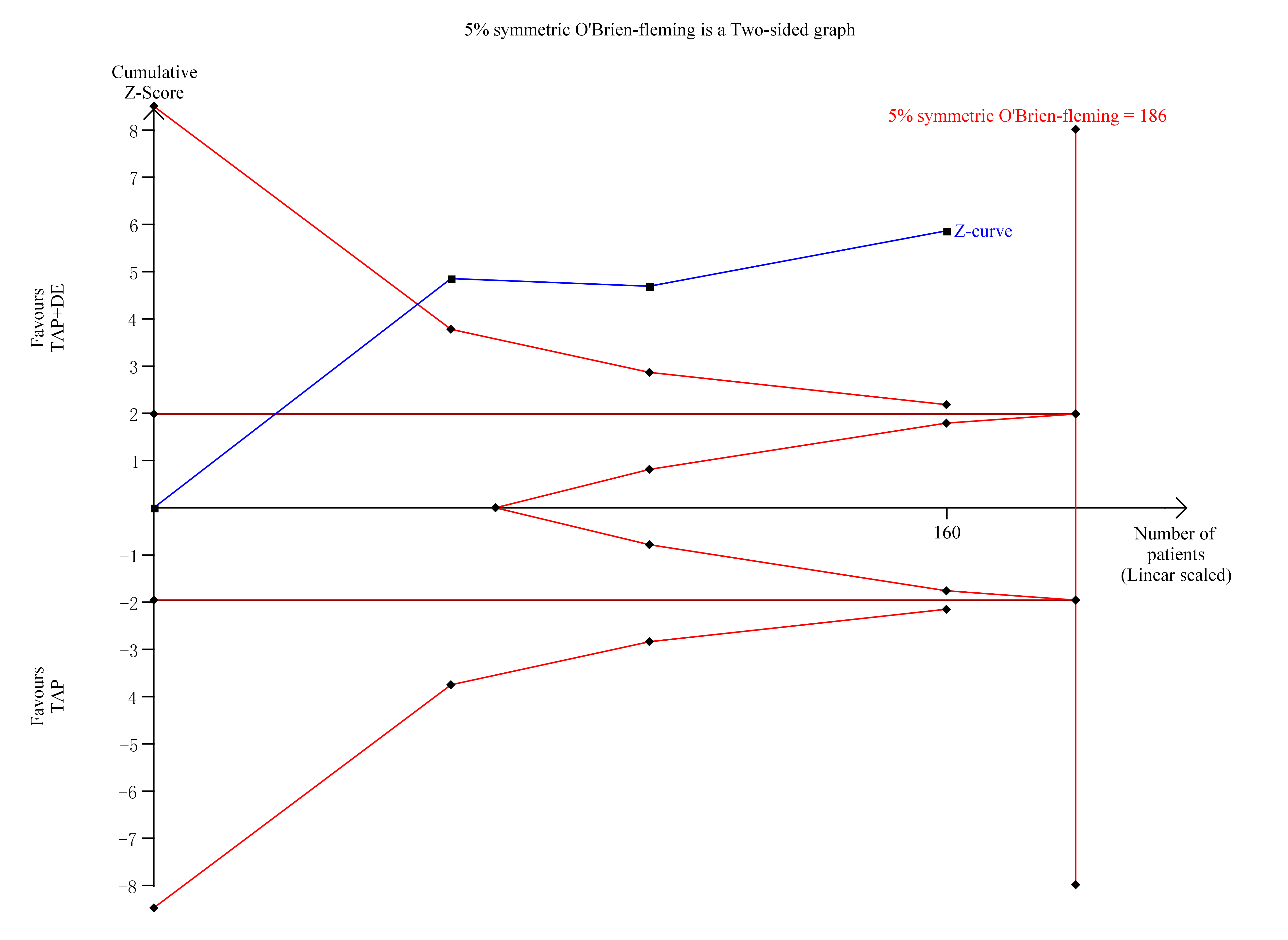

Supplement: S3 Fig — (PNG) [file pone.0198923.s004.png]

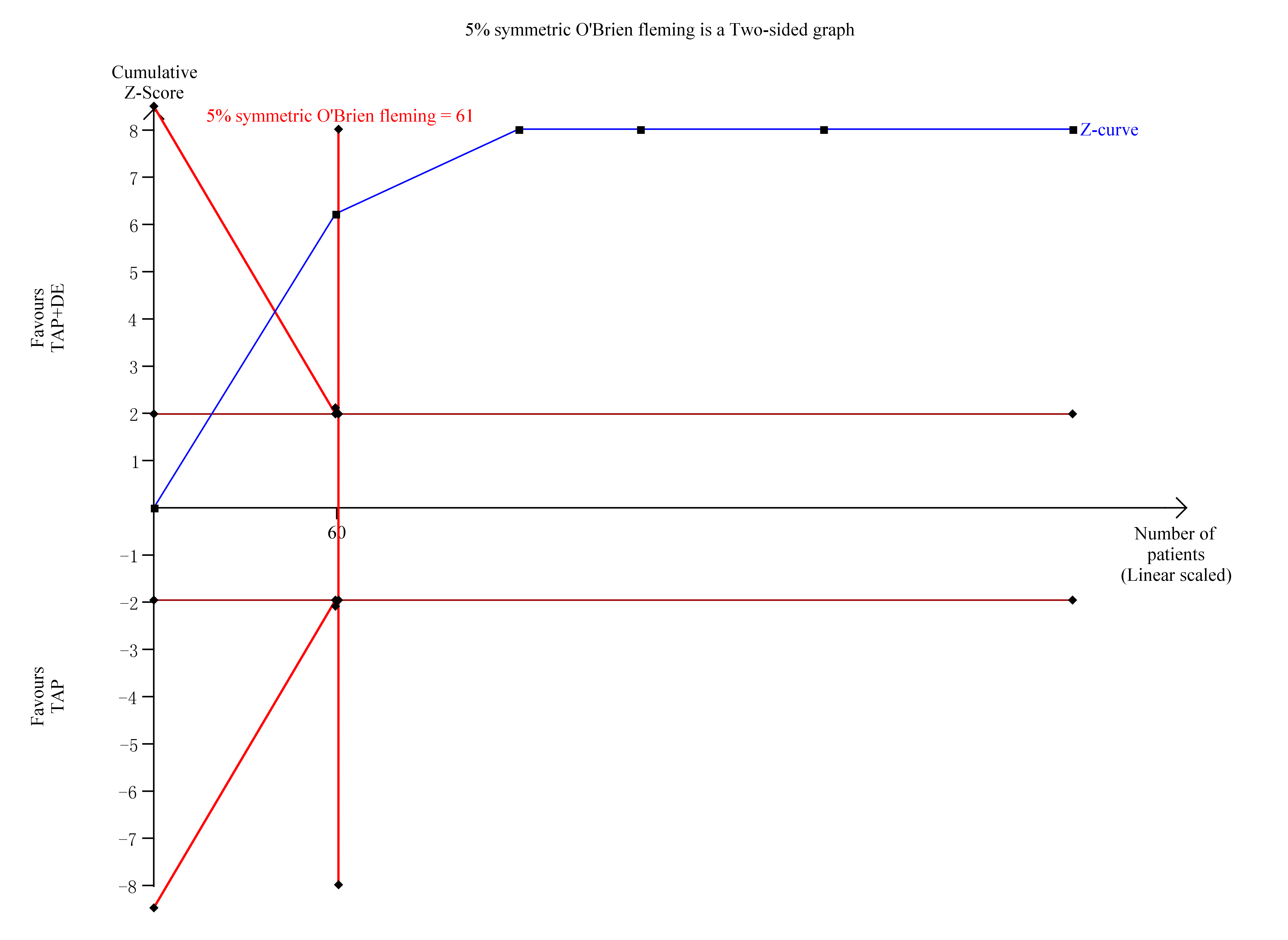

Supplement: S4 Fig — (PNG) [file pone.0198923.s005.png]

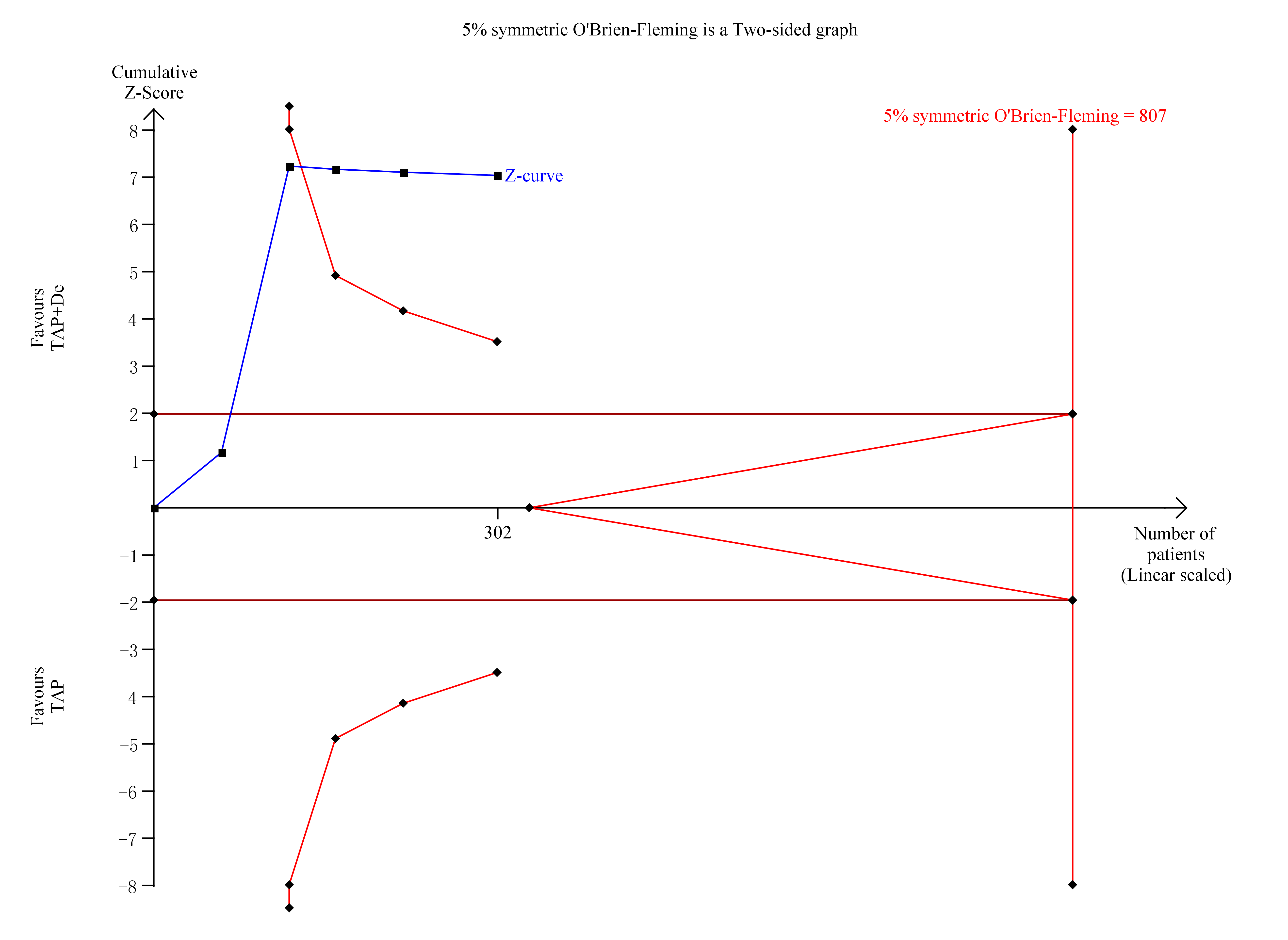

Supplement: S5 Fig — (PNG) [file pone.0198923.s006.png]

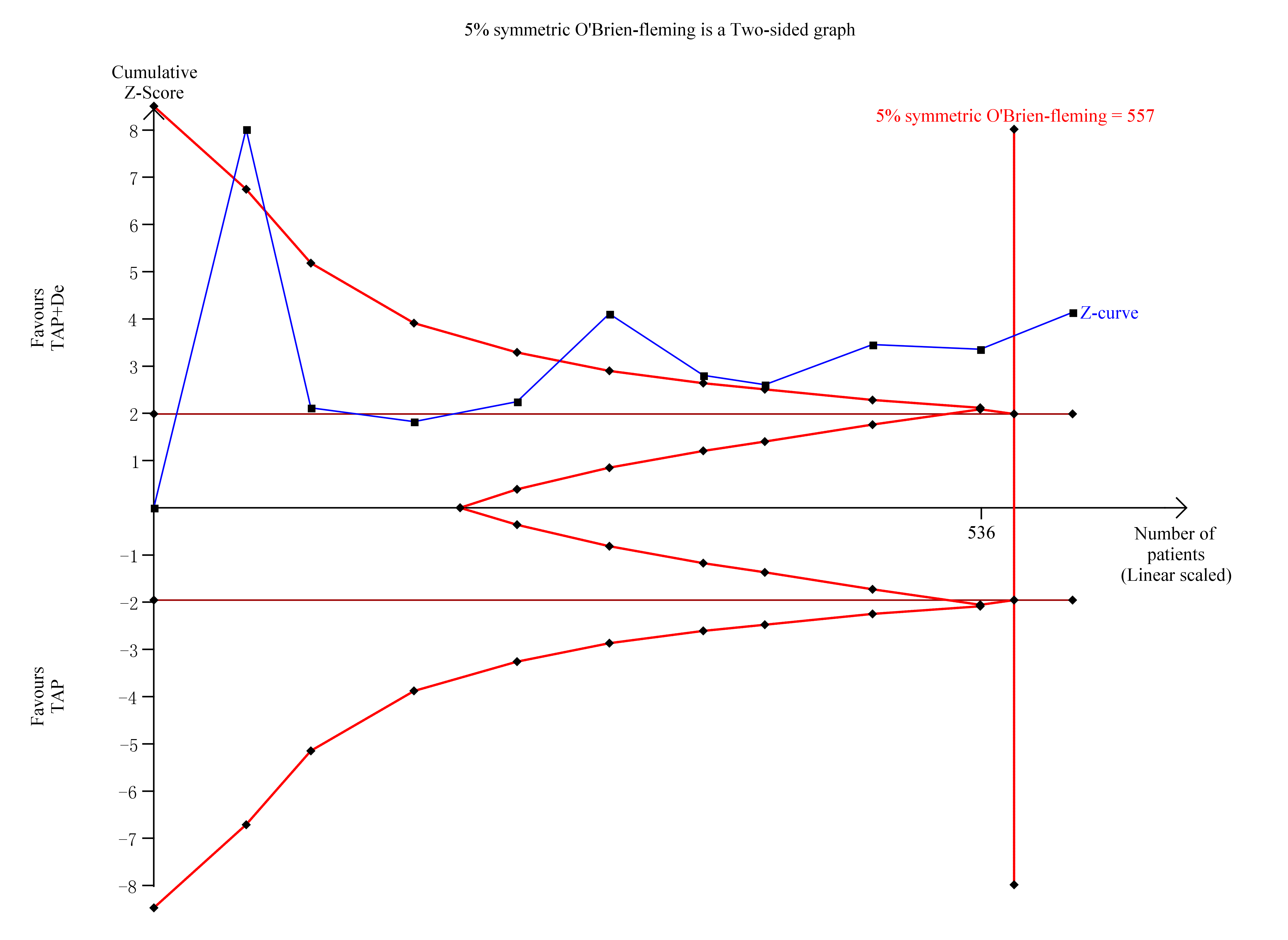

Supplement: S6 Fig — (PNG) [file pone.0198923.s007.png]

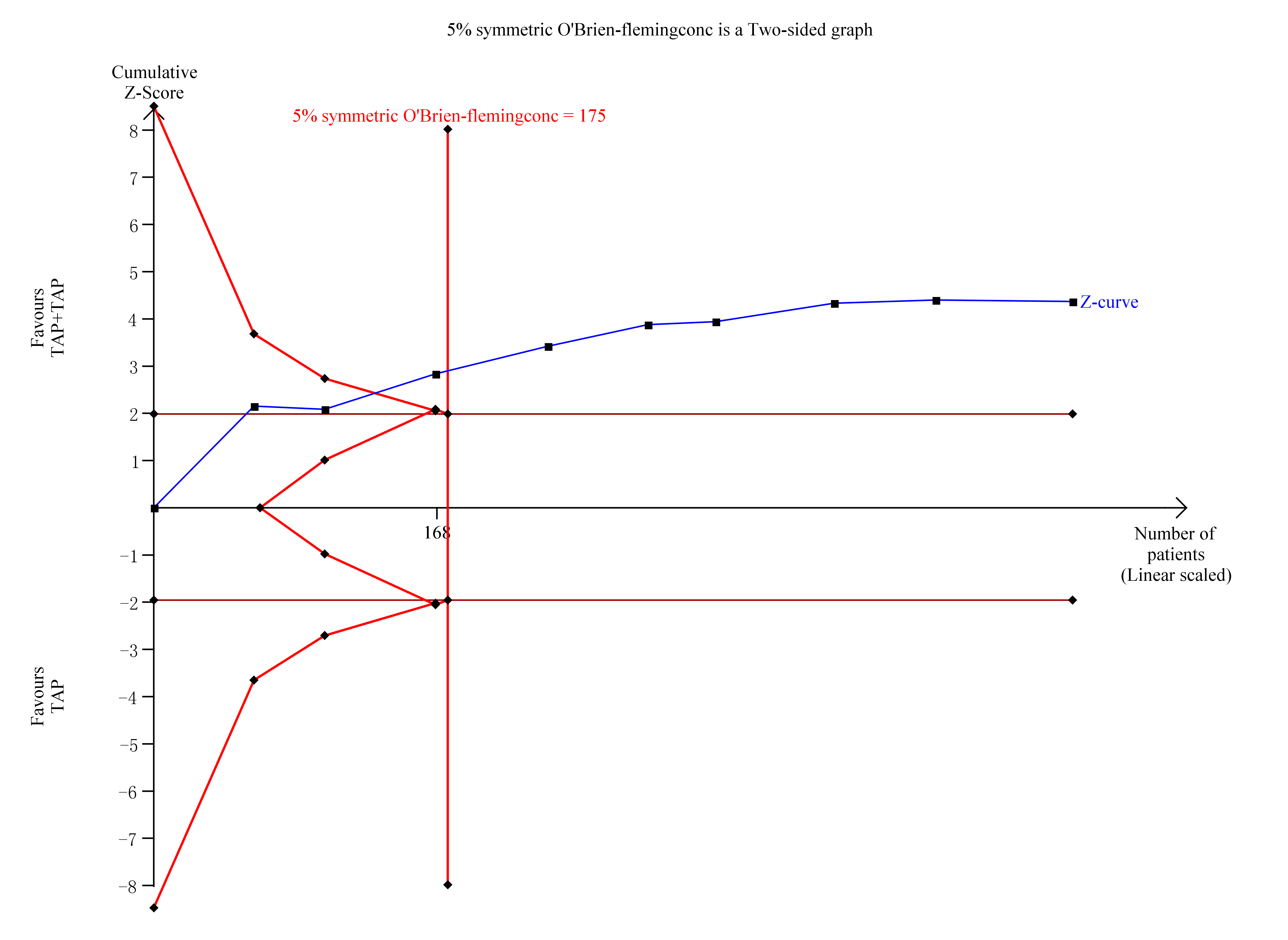

Supplement: S7 Fig — (PNG) [file pone.0198923.s008.png]
